# A new interdisciplinary perspective in the design of early evaluation and intervention programs for children with visual impairment

**DOI:** 10.3389/fped.2025.1596264

**Published:** 2025-06-13

**Authors:** Elena Mercuriali, Chiara Ceccato, Francesca Incagli, Giulia Berto, Agnese Suppiej, Maria Eleonora Reffo

**Affiliations:** ^1^ Robert Hollman Foundation, Padova, Italy; ^2^ Department of Medical Sciences, University of Ferrara, Ferrara, Italy

**Keywords:** visual impairment & blindness, early interdisciplinary evaluation, family-centred approach, child, development

## Abstract

Vision plays a crucial role in children's early development. Many studies have demonstrated that disturbance of vision affects a child's developmental trajectory, especially if it occurs early in life. The current report introduces an innovative perspective concerning the evaluation of infants and toddlers with visual impairment within a family-centred, interdisciplinary framework. It emphasizes that a collaborative, multi-professional framework is crucial for understanding and addressing the complex needs of children with visual impairment and their families, particularly during the critical early stages of life. The article presents a six-week evaluation program for children with visual impairment, aged 0–24 months, which has been implemented over the past fifteen years at the Robert Hollman Foundation, Italy. This program integrates professionals from various fields, including paediatric ophthalmology, neurology, psychotherapy, and rehabilitation therapy, to comprehensively assess the child's visual and developmental functioning. Central to this approach is the active involvement of caregivers, who are informed, engaged and empowered in relation to their child's needs. The report argues that an interdisciplinary and family-centred evaluation framework is essential for providing tailored support and facilitating effective early intervention, ultimately improving care outcomes for children with visual impairment.

## 1 Background

Timely evaluation of children with disabilities is essential to identify children's needs and strengths, ensure access to services and provide individualized treatments (1). Caregivers are key partners in early evaluation and intervention processes, and should be informed, involved, and empowered regarding to their child's developmental needs and resources (2, 3). Vision plays a crucial role in child's health, development, social experience and learning (4). Notably, lack of, or disturbed vision, especially when occurring early in life, dramatically influences a child's developmental trajectory (5–10) and early parent-child interactions (3, 11). Therefore early interdisciplinary evaluation of these children is needed to support their growth, prevent developmental delays secondary to vision impairment, enhance positive child-caregiver interactions and sustain parental wellbeing.

To support the child and family during the first few years of life, the evaluation process should be timely, clinically orientated and interdisciplinary. An interdisciplinary evaluation is a collaborative process where different disciplines of health professionals integrate their perspectives to develop a comprehensive picture of the child's and family's functioning (12). Clinicians are inter-dependent, and their perspectives are integrated from the very beginning of the evaluation process (13). Moreover, the evaluation with family involvement often provides the first step of intervention setting habilitation goals and enabling caregivers to understand and accept their child's diagnosis and developmental needs (14).

## 2 Aim

The present report aims to introduce a novel perspective on the role of interdisciplinary team in the evaluation of children with Visual Impairment (VI). We also aim to show that describing an interdisciplinary perspective, structured within a family-centred framework, allows for the development of evaluation processes that themselves constitute the first step of intervention (14). To illustrate the application of an interdisciplinary approach within a family-centred framework, we present an evaluation program designed for children with VI, aged 0–24 months, and their families. This program has been implemented over the past 15 years at the Robert Hollman Foundation (RHF), a local clinical centre for children with VI and their families in Italy.

## 3 The setting of the Robert Hollman Foundation

The RHF is a private non-profit organization that has been dedicated to supporting children with VI, from birth to 14 years of age, since 1979. The mission of RHF is to provide timely evaluation and intervention programs to promote all areas of children's development and to support the family system with an interdisciplinary and multi-dimensional approach involving professionals from different contexts (e.g., hospitals, re-habilitation centres, schools and services for leisure time). The RHF's approach has been highly valued by parents (15). Over the last 45 years, the RHF has been supporting 3,351 children from 0 to 14 years and their families (the number is updated as of May 2024). The clinical expertise with children with VI and the knowledge across professional fields (medical, rehabilitative, psychological, and educational) have contributed to the development of various interdisciplinary and family-centred evaluation and intervention programs. In this report, we present one such evaluation program.

## 4 The early interdisciplinary evaluation program

### 4.1 Key points

In the sections below, we outline the theoretical pillars that underpin the interdisciplinary approach developed for the care of children with VI. These key elements, the interdisciplinary team, reflective practice, and the involvement of caregivers (i.e., family-centred service), serve as guiding principles for all clinical processes, from assessment to intervention.

#### 4.1.1 Interdisciplinary team

The interdisciplinary team is a multi-professional body composed of different practitioners specialized in VI and neurodevelopmental disorders including a paediatric ophthalmologist, child neurologist, psychotherapist, orthoptist, rehabilitation therapist, speech therapist, and educator. Depending on diagnostic questions and clinical needs, professionals are variably involved throughout the evaluation process. The interdisciplinary approach implies that clinicians co-observe, co-evaluate the child, and regularly meet to integrate their perspectives (13, 16). Indeed, all the team members share the responsibility of the evaluation and intervention plan, and of the communication with the family. This approach differs from a multi-disciplinary model, where professionals mainly work in parallel and usually meet to discuss case problems. Such interdisciplinary work implies a high degree of commitment, communication, cooperation and trust among the members of the team, who develop skills across different professional fields (13). Although an interdisciplinary approach requires high support at the organizational and institutional level (16), it ensures the integration of different disciplines (e.g., medical, psychological, habilitative, educative) to develop a comprehensive picture of the child and family's functioning. Another advantage of an interdisciplinary group involving the psychotherapist, is that caregivers are also emotionally sustained to develop healthy coping strategies and positive parent-child interactions (14). This is particularly important to give tailored recommendations that take into account family resources in the here and now.

#### 4.1.2 Reflective practice

Reflective practice is a clinical tool that fosters critical insight within the interdisciplinary team. It enables professionals to frame thoughts, emotions, and uncertainties that may otherwise remain unspoken, allowing for the emergence of meaningful, previously unasked questions (17, 18). At the RHF, reflective practice serves as a structured space, facilitated by the psychotherapist, where the team can engage in deep, collaborative reflection to develop a comprehensive picture of the child's and family's functioning. Reflective practice becomes a meaning to integrate the clinical observations with the lived experience of the family, the child and the clinicians involved. Through this continuous and reflective dialogue, the team is better equipped to tailor communication with the family taking into account explicit and implicit concerns raised during the sessions. Lastly, in complex and emotionally charged clinical contexts, reflective practice acts as a protective factor for team members' emotional resilience, and ensures ethical, thoughtful care (19).

#### 4.1.3 Involvement of caregivers

Caregivers are continuously involved during the evaluation process. Clinicians sustain them in the observation and understanding of their child's behaviour to identify, without judgement, their child's needs and strengths and develop effective ways of interaction. The engagement of caregivers in the evaluation process is essential for the development of a readiness in the understating of their child's diagnosis, functioning and in accepting the recommendations for future interventions (20). Caregivers' involvement in the observation and direct play with their child, fosters the development of positive parent-child interactions that have been demonstrated to decrease parental stress and enhance the perception of a supportive environment (3). Furthermore, caregivers are encouraged by the psychotherapist to express their concerns and inner experiences with empathic listening (21). Such a family-centred approach enables the planning of tailored and shared intervention programs, in which the child and family's resources are equally considered and their needs identified (22).

### 4.2 Main goals and setting

The main goals of our early inter-disciplinary evaluation program designed for children with VI from 0 to 24 months are summarized in Table 1. The program is structured into six sessions, with one session per week. The multiple-week structure of the evaluation program allows professionals to monitor developmental changes during a period of rapid growth and neural plasticity, such as the first two years of life, and to establish goals and strategies accordingly (23). Caregivers are always present during the evaluation to favour their understanding of the child's visual functioning and to promote their active participation and shared decision-making.

**Table 1:** Main goals of the evaluation program.

✓ Evaluation of the child's visual functioning and developmental profile ✓ Identification of the resources available to the child and family to rely on ✓ Empowerment of caregivers through support in understanding their child's behaviour and needs, and in developing new ways of interaction ✓ Tailored communication to support comprehension of the diagnosis and clinical profile

The evaluation program begins with clinical screening performed by the psychotherapist, usually by phone, during which the family's referral questions and expectations are collected. Afterwards, the sessions are scheduled with the family. Each session lasts two hours. One hour and half is dedicated to the evaluation, while the last thirty minutes are devoted to the discussion within the interdisciplinary team. This prolonged time (i.e., two hours) is conceived to meet the needs of an infant with VI (e.g., fatigue, sleep, diaper changes etc.). For instance, if the child is hungry and needs to be fed, the scheduled activities are postponed, and the team observes mother-child interactions and shares with the family, facilitators to be used in this context (e.g., adding white-black stripes on the baby's bottle). Flexibility among sessions is possible thanks to the multiple week structure of the evaluation program and to the collaborative attitude of the members of the interdisciplinary team. For instance, if the child is asleep or conversely, cries during the visual function evaluation on the first session, the assessment is shifted to the following one.

Each session takes place in an adapted space. The environment is enriched by facilitators according to the needs of children with VI, such as high-contrast objects, multi-sensory materials, adaptable over-lighting, big pillows, and sofas where the child can safely move and play. The activities proposed by professionals are always thought to be relevant for diagnostic, habilitative and relational purposes. For instance, the rehabilitation therapist may use a flashlight to illuminate an object (visual stimulation), then he/she can move the light to illuminate his/her own face, or the face of caregivers (relational input). At the end of each session, the interdisciplinary team meets to discuss what they have just observed. The psychotherapist supports the team members in integrating their clinical observations with their lived experiences by using reflective practice. It is noteworthy that, in this novel approach, a significant role is given to the psychotherapist. They are the professionals who establish contact with the family, coordinate the different sessions and support caregivers in understanding the child's behaviour in the here-and-now of the assessment, using a strength-based approach (24). For instance, it is common to observe that children with severe VI or blindness, when called by their name, orient their ears toward the sound source as a strategy to focus attention on relevant stimuli (i.e., acoustic input). This behaviour is commonly mis-interpreted by parents as a refusal signal from their child. In this case, the psychotherapist guides the family in correctly interpreting their child's communication signal, transforming an apparent behaviour of rejection into an opportunity to learn and to positively interact with their child. The psychotherapist plays also a pivotal role in supporting parents to cope with their inner experience (21).

The goals, the professionals involved, and the main instruments and strategies used for each session are reported in Supplementary Table S1.

### 4.3 Visual function evaluation

The visual function evaluation is performed during the first session by the orthoptist (see Supplementary Table S1). The psychotherapist, rehabilitation therapist, and caregivers are also present. The main aim of the visual function assessment of children from 0 to 24 months is the evaluation of oculo-motor function, visual attention, visual acuity, contrast sensitivity, visual field and light adaptation using observation and behavioural techniques (e.g., preferential looking techniques). This is usually a distressing moment for caregivers, since it is often the first-time they have received information on *how* their child sees. Due to the relevance of this moment, at the end of the assessment, the interdisciplinary team meets without caregivers to tailor the communication considering the characteristics of each family. This reflexive time is relevant to gradually guide caregivers in the understanding of their child's visual functioning and to cope with this emotionally challenging moment.

### 4.4 Developmental profile evaluation

During the second, fourth and fifth sessions, a central role is given to the developmental profile evaluation performed by the rehabilitation therapist (see Supplementary Table S1). The aim of these sessions is to assess the overall child's functioning and the interplay among developmental areas, specifically sensory-motor, cognitive, socio-emotional and communication skills. Functional vision is evaluated by observing whether and how the child uses vision to interact with professionals and caregivers and to explore the surrounding environment under real-life conditions (25). Additionally, according to children's developmental age and clinical profile, the therapist observes visual attention, visuomotor control, eye-hand coordination, motion perception, recognition of spatial relations, visual planning, and object recognition, during activities of free playing. The functional use of available vision and multi-sensory integration is then promoted with tailored strategies, postures and multi-sensory materials (e.g., high contrast object). The psychotherapist observes the therapist-child interaction and guides caregivers in understanding the child's visual responses and behaviours with a strength-based and developmental approach. At the end of each session, the most significant activities are also shared with the family to sustain the generalization of child's achievements at home.

### 4.5 Ophthalmological evaluation

The ophthalmological evaluation is performed during the third session (see Supplementary Table S1). The ophthalmologist visits the child at the presence of the whole team in the medical room or in the same setting of the evaluation program, accordingly to the child's needs. After the physician has read the medical documentation, each member of the team reports observations and evaluations from their own professional perspective. This helps to share insights on the child's visual functioning and on the family's resources based on what was observed in the previous sessions. The evaluation starts with the orthoptist repeating the visual function assessment in collaboration with the ophthalmologist, who afterwards performs the ocular anterior segment examination, autorefraction before and after cycloplegia to seek for refractive errors, and fundus oculi examination under mydriasis. During the cycloplegia waiting time, the interdisciplinary team and the ophthalmologist discuss visual function results and the aetiology of VI. Moreover, the interdisciplinary team and the physician, supported by the psychotherapist, share insights on the family's characteristics, resources, implicit and explicit questions, and expectations. This allows for tailored communication that takes into account the caregivers' emotional state and supports them in understanding and accepting their child's visual profile, particularly when the information is first provided. The physician informs caregivers concerning the child's visual function profile, ophthalmological diagnosis, spectacle correction and/or optical filters if needed, and provides guidance on further medical investigations or interventions according to the child's clinical picture. After spectacle correction, an additional assessment is scheduled to asses changes in visual functions as well as in functional vision.

### 4.6 Neurological evaluation

The neurological examination is conducted by a paediatric neurologist, when indicated by diagnostic or clinical needs, during the third session. This is particularly relevant since VI impacts on child's global health and development, especially if it is congenital (26). Additionally, severe VI and blindness often occur in the setting of multisystem neuro-metabolic or genetic disorders with onset in the first year of life (27, 28). In these children, Cerebral Visual Impairment (CVI) is the first cause of VI (29). Therefore, in acquired or genetic disorders at risk of neuro-visual system involvement, such as the case of extreme prematurity, cerebral palsy, syndromic disorders, and traumatic brain injury, CVI is also investigated. For these reasons, the paediatric neurologist, with expertise in neuro-ophthalmology, has to be part of the multiprofessional team, to ensure the best and timely care for these children and their family (30). In cases of retinal and optic nerve disorders, as well as neurological conditions involving cerebral visual areas, the paediatric neurologist, in collaboration with the ophthalmologist, prescribes visual-electrophysiological exams, including electroretinography and/or visual evoked potentials (31, 32). Guidance for further medical examination (e.g., genetics or metabolic investigations) is also provided.

As during the ophthalmological evaluation, the interdisciplinary team firstly share with the paediatric neurologist insights on child's functioning and family's characteristics. Then, in the same setting of the evaluation program, the physician observes the child interacting with the rehabilitation therapist, and afterwards performs the neurological examination (see Supplementary Table S1). After clinical assessment, the interdisciplinary team and the child neurologist make a shared plan on how to tailor communication accordingly to family functioning. Then, the paediatric neurologist describes to caregivers the neurological results and the medical diagnosis within a comprehensive picture of the child's functioning with a strength-based approach.

### 4.7 The feedback session

During the last session, the interdisciplinary team shares with the caregivers a comprehensive picture of their child visual and developmental profile and gives them practical suggestions that can be integrated into their daily routines. The feedback is based on what professionals have done with the child and what caregivers have actively observed. This ensures that caregivers can more effectively understand and generalize facilitators and strategies to other contexts. Professionals provide guidance on establishing an accessible home environment by recommending the use of tactile and auditory cues, high contrast, appropriate lighting, and consistent spatial organization. Core suggestions on visual, and multi-sensory characteristics of playing materials and daily life objects are provided, too. Additionally, tailored strategies to support the child's daily functioning and to promote more effective and positive interactions are shared. These include recommendations such as postures that enhance visual performance, verbal anticipation, tactile guidance, gradual exposure to new activities, and strategies to prevent fatigue. The information is given verbally, so that caregivers can ask further questions. Then a written summary, in the form of a clinical report, is sent to the family in order to be shared with other health, educational and social care services. The recommended adaptations and strategies are also summarized in a user-friendly document, named Hollman Facilitations (HFs). This tool has been extensively described in a recent publication (33).

The goal of this session is to provide information that is targeted on family's needs and resources. A tailored communication supports caregivers to emotionally cope with the challenge of being a parent of a child with disability, to search for specialized services and facilities, and to access health, social and financial benefits (20).

## 5 Summary

The present report describes a novel approach to the early evaluation of children with VI within a local clinical service for children with VI and their families in Italy. We outline the strengths of an interdisciplinary approach which is based on the inter-dependence among team members who co-observe and co-evaluate the child throughout the period of the assessment. This deeply collaborative work allows for the development of a comprehensive picture of the child's developmental and visual profile, integrating medical, re-habilitative, and psychological dimensions. The family-centred nature of this approach implies the involvement of caregivers, that plays an active role during the evaluation process. Parents are considered by the team as partners of the intervention and sustained in the observation and identification of their child's characteristics with a strength-based and non-judgmental attitude (34). By supporting caregivers in understanding their child's characteristics through a strength-based approach and helping them to find new ways of interaction, the early, interdisciplinary evaluation process itself can become the first step of the intervention (14). A longitudinal study to assess the effects of this early interdisciplinary evaluation program, designated as Robert Hollman Foundation Early Support Path (RHF-ESP), on parental stress, children's adaptive behaviour, and on parent-child interactions, is ongoing (https://doi.org/10.17605/OSF.IO/45PV7). Furthermore, future studies should explore the generalizability of the program to other settings, such as hospitals or primary care services.

## Data Availability

The original contributions presented in the study are included in the article/Supplementary Material, further inquiries can be directed to the corresponding author.
