# Supplementary material for: A new interdisciplinary perspective in the design of early evaluation and intervention programs for children with visual impairment
Source: Front Pediatr. 2025 Jun 13;13:1596264. doi: 10.3389/fped.2025.1596264 (PMC12202372; doi:10.3389/fped.2025.1596264)
Supplement: Supplementary file 1 [file Table1.docx]

| **Supplementary Table 1.** Design, professionals involved, aims, strategies and instruments of the early interdisciplinary evaluation program. | | | |
| --- | --- | --- | --- |
|  | **Professionals** | **Aim** | **Strategies/instruments** |
| **First session**  *Main aim: Visual function evaluation* | Orthoptist | - Basic visual function evaluation:   - Oculo-motor function (fixation, strabismus, nystagmus, saccades/smooth pursuit eye movements)   - Visual attention   - Resolution visual acuity   - Contrast sensitivity   - Visual field   - Light adaptation | - Spontaneous visual behavior observation - Observation of oculomotor function using high-contrast objects - Visual attention shift paradigm (attention shift with or without competition) - Grating acuity (i.e., Teller Acuity Cards II; 37) - Hiding Heidi test - Behavioral confrontation techniques for visual field |
|  | Rehabilitation therapist | - Observation of child’s visual behavior either when the child is held in caregiver’s arms, in the postural system or on the mat - Observation of caregiver’s handling and holding strategies | - Maneuvers of child’s handling and holding in support of the visual function evaluation |
|  | Psychotherapist | - Collection of the child’s medical history (anamnesis) - Development of a participatory and trust relationship with caregivers - Parental support | - Emphatic and non-judgmental listening (22) - Participant observation (38) - Dialogue with caregivers to collect child’s medical history, and to listen to their questions, worries, expectations, and inner experiences - Parental Stress Index (PSI; 39) is a self-report questionnaire often given to caregivers at the beginning and at the end of the assessment to monitor parental burden |
|  | Interdisciplinary equipe | - Integration of professionals’ observations to develop a holistic view of the child-family system - Reflection on and sharing of the emotions that professionals experience during the evaluation - Design the setting for the next session: environment, timing, activities and adapted materials to propose | - Reflective practice: the psychotherapist supports the equipe to use the countertransference information, not to interpret, but as evidence of the emotional-relational functioning of the family itself - Writing of the clinical report |
| **Second session**  *Main aim: Developmental profile evaluation* | **Professionals** | **Aim** | **Strategies/instruments** |
|  | Orthoptist | - Usually not present |  |
|  | Rehabilitation therapist | - Observation and evaluation of child’s behavior, such as: neuro-motor skills (e.g., head control, rolling etc.), stress signals, consolability, interaction with familiar and non-familiar adults - Observation of functional vision: visual attention, visuomotor control, eye-hand coordination, motion perception, recognition of spatial relations, visual planning and object recognition - Observation and evaluation of child responses to visual and non-visual stimuli (e.g., tactile, auditory) in a facilitated environment (e.g., *Does the visual behavior of the child differ from the one observed in a more structured setting, such as the one during the Teller Acuity Cards testing? Is visual attention enhanced by a facilitated environment and/or by multi-sensory objects?*) - Sharing of activities and materials with caregivers to be used with the child at home | - Neonatal Behavioral Assessment Scale (NBAS; 25); - Using of facilitated activities at the level of postures (e.g., baby bouncer), objects (e.g., multi-sensory materials) and environment (e.g., adapted lighting) |
|  | Psychotherapist | - Anamnesis and dialogue with caregivers - Sustain caregivers in developing readiness in understanding their child’s behavior - Parental support | - Emphatic and non-judgmental listening - Translation of the child’s behavior in the here and now of the assessment to support caregivers’ understanding with a strength-based approach (25) |
|  | Interdisciplinary equipe | - See description in First session | - See description in First session |
| **Third session**  *Main aim: Ophthalmological evaluation and Neurological examination ^a^* | **Professionals** | **Aim** | **Strategies/instruments** |
|  | Orthoptist | - Basic visual function assessment (in collaboration with the ophthalmologist) | - See strategies and instruments described in First session |
|  | Ophthalmologist | - Ophthalmological assessment - Communication of visual function evaluation and ophthalmological diagnosis - Spectacles, optical filter prescription - Recommendations for further medical investigations (e.g., electroretinography, visual evoked potentials, ophthalmological imaging) or interventions | - Anterior segment evaluation - Autorefraction before and after cycloplegia (refractive errors) - Fundus oculi evaluation under mydriasis |
|  | Child neurologist | - Child's behavioral observation - Pediatric neurological examination - Clarification of the child’s medical picture - Recommendations for further medical investigation (e.g., metabolic and genetic investigations) - Interpretation of electroretinography and visual evoked potentials and other available investigations from the neurophysiological/neuro- ophthalmological perspective and within the global medical diagnosis | - General evaluation of: - Orientation and behavior (e.g., consolability, attention to the environment, auditory and visual attention) with a specific focus on abnormal visual behaviors and oculomotor function - cranial circumference (%ile), comparison with height and weight centiles, dysmorphic features, skull, face, skin, limb, organomegaly - Tone of limbs, neck, trunk, strength - Spontaneous movements (e.g. general movements) - Reflexes |
|  | Interdisciplinary equipe | - Sharing with physicians the child’s medical history, professionals’ observation on child’s clinical profile and on family functioning (e.g., expectations and fears) - Discussion on how to communicate to the family the ophthalmological and neurological results - Emotional support of caregivers (by the psychotherapist) | - Reflective practice - Trust and collaborative attitude among all professionals |
| **Fourth session**  *Main aim: Developmental profile evaluation* | **Professionals** | **Aim** | **Strategies/instruments** |
|  | Orthoptist | - Usually not present |  |
|  | Rehabilitation therapist | - Interaction with the child - Observation and evaluation of gross motor skills and postures, sensory-motor adaptation to the therapist proposals, child’s stress signals, consolability, separation with caregivers - Sharing with caregivers adapted materials and activities that can be performed at home | - Playing activities with the child - Use of facilitated materials (e.g., high contrast objects), activities and environment (e.g., room lighting) - Postural stability |
|  | Psychotherapist | - Emotionally support the family to share what emerged during the ophthalmological and neurological examination, facilitating the expression of not-asked questions and their inner experience - Support the family in their understanding of the impact of VI in their child daily life - Sustain the parental sense of self-efficacy and empowerment - Sustain positive parent-child interaction and relationship | - Emphatic and non-judgmental listening - Strength based approach: focus on family’s and child resources - Translation of the child’s behavior in the here and now of the assessment to support caregivers’ understanding with a strength-based approach (25) |
|  | Interdisciplinary equipe | - See description in First session | - See description in First session |
| **Fifth session**  *Main aim: Developmental profile evaluation* | **Professionals** | **Aim** | **Strategies/instruments** |
|  | Orthoptist | - Usually not present |  |
|  | Rehabilitation therapist | - Repetition and consolidation of what was done in the previous session - Active involvement of caregivers in the interaction with the child | - Playing activities with the child - Use of facilitated materials (e.g., high contrast objects), activities and environment (e.g., room lighting) - Postural stability |
|  | Psychotherapist | - Emotionally support the family to share what emerged during the ophthalmological and neurological examination, facilitating the expression of not-asked questions and inner experience - Support the family in their understanding of the impact of VI in their child daily life - Sustain the parental sense of self-efficacy and empowerment - Sustain positive parent-child interaction and relationship | - Positive attitude - Strength-based approach - Translation of the child’s behavior in the here and now of the assessment to support caregivers’ understanding with a strength-based approach (25) |
|  | Interdisciplinary equipe | - See description in First session | - See description in First session |
| **Sixth session**  *Main aim: Tailored communication of the results of the evaluation to the family* | **Professionals** | **Aim** | **Strategies/instruments** |
|  | Orthoptist | - Evaluation of some item of the basic visual function assessment with the focus on child’s achievements (strength-based approach) - Answer to caregivers' questions concerning the ophthalmological evaluation | - See description in First session |
|  | Rehabilitation therapist | - Description of child’s functioning and achievements, highlighting how vision is strictly connected to all areas of development (sensory-motor, cognition, emotion, relation and interaction) - Guidance to sustain child’s daily functioning and development at home, such as environmental adaptations, visual and multisensory features of playing materials and daily objects, gradual exposure to new activities | - Hollman Facilitations (HFs; 34) |
|  | Psychotherapist | - Describe child’s resources and achievements with a strength-based approach - Empower caregivers regarding their child management - Provide parental support to emotionally cope - Support caregivers in activating an early intervention network among professionals differently involved in their child’s daily life (e.g., pediatrician, healthcare professionals, teachers etc.) | - Emphatic and non-judgmental listening - Strength based approach: focus on family’s and child resources |
| ^a^ The neurological examination is conducted when required, based on diagnostic and clinical needs. | | | |
